# The presence of a booster phenomenon among contacts of active pulmonary tuberculosis cases: a retrospective cohort

**DOI:** 10.1186/1471-2458-7-38

**Published:** 2007-03-19

**Authors:** Cristiane G Salles, Antonio Ruffino-Netto, Jose R Lapa-e-Silva, Afranio L Kritski, Michelle Cailleaux-Cesar, Fernanda C Queiroz-Mello, Marcus B Conde

**Affiliations:** 1 Instituto de Doencas do Torax/Hospital Universitario Clementino Fraga Filho – Universidade Federal do Rio de Janeiro. Rua Rodolpho Rocco, 255/3° andar, SME da Pneumologia. Rio de Janeiro, RJ, Cep 21941-913, Brazil.; 2 Faculdade de Medicina de Ribeirão Preto – Dept° de Medicina Social – Av. Bandeirantes, 3.900 Ribeirao Preto Ribeirão Preto – SP – Cep: 14.049-900, Brazil

## Abstract

**Background:**

Assuming a higher risk of latent tuberculosis (TB) infection in the population of Rio de Janeiro, Brazil, in October of 1998 the TB Control Program of Clementino Fraga Filho Hospital (CFFH) routinely started to recommend a two-step tuberculin skin test (TST) in contacts of pulmonary TB cases in order to distinguish a boosting reaction due to a recall of delayed hypersensitivity previously established by infection with *Mycobacterium tuberculosis (M.tb) *or BCG vaccination from a tuberculin conversion. The aim of this study was to assess the prevalence of boosted tuberculin skin tests among contacts of individuals with active pulmonary tuberculosis (TB).

**Methods:**

Retrospective cohort of TB contacts ≥ 12 years old who were evaluated between October 1^st^, 1998 and October 31^st ^2001. Contacts with an initial TST ≤ 4 mm were considered negative and had a second TST applied after 7–14 days. Boosting reaction was defined as a second TST ≥ 10 mm with an increase in induration ≥ 6 mm related to the first TST. All contacts with either a positive initial or repeat TST had a chest x-ray to rule out active TB disease, and initially positive contacts were offered isoniazid preventive therapy. Contacts that boosted did not receive treatment for latent TB infection and were followed for 24 months to monitor the development of TB. Statistical analysis of dichotomous variables was performed using Chi-square test. Differences were considered significant at a p < 0.05.

**Results:**

Fifty four percent (572/1060) of contacts had an initial negative TST and 79% of them (455/572) had a second TST. Boosting was identified in 6% (28/455). The mean age of contacts with a boosting reaction was 42.3 ± 21.1 and with no boosting was 28.7 ± 21.7 (p = 0.01). Fifty percent (14/28) of individuals whose test boosted met criteria for TST conversion on the second TST (increase in induration ≥ 10 mm). None of the 28 contacts whose reaction boosted developed TB disease within two years following the TST.

**Conclusion:**

The low number of contacts with boosting and the difficulty in distinguishing boosting from TST conversion in the second TST suggests that the strategy of two-step TST testing among contacts of active TB cases may not be useful. However, this conclusion must be taken with caution because of the small number of subjects followed.

## Background

The tuberculin skin test (TST) is used to determine if a person has been infected by *Mycobacterium tuberculosis *[1]. However, response to *M. tuberculosis *antigens may wane over time in an individual with latent tuberculosis (TB) infection or who is exposed to nontuberculous mycobacteria, including BCG vaccine. Re-exposure with tuberculin may result in an anamnestic-like response of the cellular immune system, resulting in a subsequent positive reaction on repeated skin testing. The use of serial TSTs to detect new instances of infection among high risk populations such as healthcare workers or contacts of pulmonary TB cases with negative TST in the initial evaluation has presented a new question: is an increase in the second tuberculin reaction size due to a recall of delayed hypersensitivity previously established by infection with *Mycobacterium tuberculosis (M.tb) *or BCG vaccination that has waned over the years or due to a new *M.tb *infection (conversion) [2]? Because of this issue, some centers with a high prevalence of TB utilize a two-step TST with a second tuberculin test being performed between 1 and 5 weeks after the first test [3,4]. Assuming a higher risk of latent TB infection in the general population of the city of Rio de Janeiro, with a TB incidence rate of 114/100,000 [5], in October 1998 the TB Control Program of Clementino Fraga Filho University Hospital (CFFH) of the Federal University of Rio de Janeiro (FURJ), Brazil, routinely started to recommend a two-step TST in contacts of pulmonary TB cases.

The purpose of this study was to assess the prevalence of boosted tuberculin skin test among contacts of individuals with active pulmonary TB and the importance of this strategy for a TB Control Program in a setting with a high prevalence of TB.

## Methods

All charts of TB contacts evaluated between October 1^st^, 1998 and October 31^st ^2001 were retrospectively reviewed by trained staff. Contacts with two-step TST (test 1 and test 2) and chest X-ray results described in the chart were enrolled in the study and had data recorded.

Contacts of culture positive TB cases are routinely evaluated 4–6 weeks after the start of the anti-TB treatment of the index case (using the Brazilian Ministry of Health recommended regimen of isoniazid, rifampin and pyrazinamide). The evaluation of household contacts was performed by the TB Control Program staff and included a two-step TST and a chest X-ray. The TST was applied to the forearm using the Mantoux technique and was read by health care workers trained and certificated (degree of intra-reader reliability > 95% and inter-reader reliability ≥ 80%). All testing was performed using purified protein derivative (PPD), RT23 (0.1 ml = 2 TU) (Staten Serum Institute, Copenhagen, Denmark). The initial TST (test 1) was read 2–3 days after application. Contacts with a reading on the first TST (test 1) of ≤ 4 mm induration were classified as negative [6] and were asked to return for a second TST (test 2) applied 1–2 weeks after test 1. Boosting reaction was defined as having a reaction of ≥ 10 mm on test 2, with an increase in induration of at least 6 mm related to test 1 [7,8]. Contacts without boosting were submitted to a third TST (test 3) 12 weeks after the first evaluation to evaluate TST conversion [6]. Boosting subjects (as evaluated at test 2) did not receive treatment for latent tuberculosis infection (TBLI) and were followed-up for at least 24 months to determine their TB status.

The study was approved by the Ethic Committee of Federal University of Rio de Janeiro (Brazil).

Statistical analysis of continuous variables was performed using Student's t-test and of dichotomous variables using Chi-square test. Differences were considered significant at a p < 0.05. The analysis was performed using SPSS Version 11.0.

## Results

**Figure 1:**
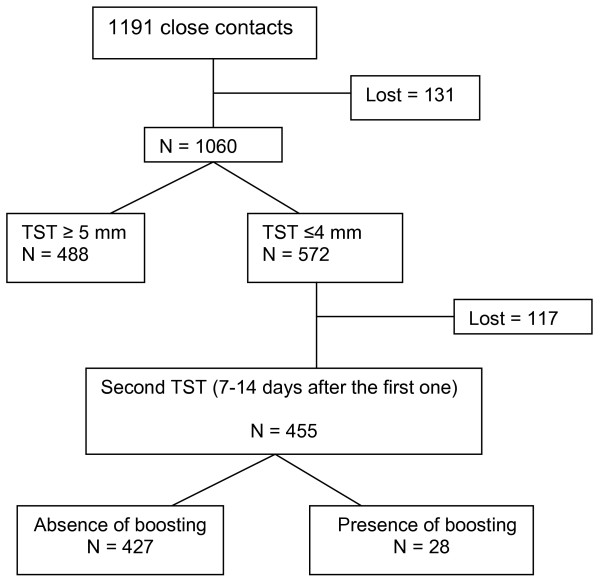
Prevalence of boosting among contacts of active pulmonary tuberculosis cases.

The study population was comprised of 1,191 identified contacts of 283 culture positive pulmonary TB index cases. In 89% (1060/1191) of these contacts an initial TST was performed and read. Figure 1 presents the flow of the study. Among 54% (572/106) of contacts with a negative initial TST (TST ≤ 4 mm), 455 had a second TST applied and read 1–2 weeks after the first test and the criteria for a "booster" phenomenon was met in 6% (28/455). Fifty percent (14/28) of contacts identified as boosting had an increase of the induration size of at least 10 mm in the second TST. In table 1 the characteristics of contacts are presented. The mean age of contacts with a "booster" phenomenon (42.3 years; ± 21.1) was higher than contacts with no "booster" phenomenon (28.7 years; ± 21.7; p = 0.001). Only 14% (4/28) of contacts were tested for HIV and all were negative. Nine percent (11/127) of contacts with no BCG scar demonstrated "booster" phenomenon compared to 5% (16/296) with BCG scar (p = 0.2). None of the 28 contacts defined by the TB Control Program as having a "booster" phenomenon developed TB disease within two years after the TST.

**Table 1:** Characteristics of 455 contacts according with the presence of boosting

	Presence of boosting	Absence of boosting	P value
Gender (male/female)	9/19	151/276	0.8
Presence of BCG scar* (yes/no)	16/11	293/127	0.2
Age (mean ± SD)	42.3 ± 21.1	28.7 ± 21.7	0,001

SD: standard deviation; * There was information about BCG vaccination in the chart of 447 contacts that underwent the second TST.

## Discussion

Six percent (28 subjects) of 455 initially negative contacts ≥ 12 years old who had a second TST applied and read within 2 weeks after the first test boosted on retesting and none developed active TB in 2 years of follow up.

Some North America guidelines, propose treatment with isoniazid for contacts of smear positive pulmonary TB with a TST ≥ 5 mm as well as for those with a tuberculin conversion demonstrated as an increase of 10 mm in the induration size of a second tuberculin test within a two year period [1,6].

The prevalence of boosting is generally correlated with the prevalence of the initial positive tuberculin reactions [2]. Gordin *et al *(1988) evaluated a large group of people in several chronic care facilities and found an initial rate of positive tests in 28% (477/1728) of the subjects and a boosting rate of 14% (158/1146). Since 46% (488/1060) of contacts in the present study were TST positive, a boosting rate higher than 6% would be expected. The finding in our sample is similar to a study performed among students in Canada that found a boosting rate of 5.2% (74/1435), however in that study the initial TST rate was 6.6% [9]. Vaccination with BCG among children less than one year of age has been obligatory in Brazil since 1976 and compliance with this policy is assumed high. The presence of BCG scar apparently did not have any influence on the incidence of a "booster" phenomenon in this series of contacts. The mean age of the subjects (28.8 years old) evaluated in this study means that BCG vaccination occurred more than 10 years before the TST may justify this finding [9].

In the TB Control Program of CFFH/FURJ the first TST of contacts is routinely done 4–6 weeks after the index case starts TB treatment and a second TST (when indicated) is done 1–2 weeks after the first test. In the current study, 50% of contacts who met criteria for "booster" phenomenon in the second TST had an increase ≥ 10 mm of induration size, which is both the Brazilian and the widely recognized criterion for identifying a tuberculin conversion case [1,2,6]. As there is a significant decrease in the infectiousness of pulmonary TB in the index case after starting anti-TB drugs, patients can generally be assumed as noninfectious to contacts after 2–4 weeks of treatment [10]. The period of time between the decrease in the infectiousness of the index case and the placement of the second TST, as well as the documented risk of infection among contacts of active TB cases makes it difficult to distinguish which contact is boosting and which one is a recent tuberculin conversion (recent infection) [11]. Two different studies have shown that among TST converters the risk for developing active TB in the first year of follow up was 8 times higher than in the subsequent seven years and that 82% of TB cases developed active disease within 2 years of infection [4,6]. None of the 28 subjects categorized as boosters in this study developed active TB within the two year follow-up period. However, the small sample size does not allow for a conclusion as to whether these subjects represented boosting phenomena instead of recent conversion, even though it is not enough to definitively undermine importance of two step TST strategy in a different clinical context. The low number of contacts of active TB cases with boosting identified over three years (6%), the cost and the time consuming beyond the difficulty in distinguishing boosting from TST conversion in the second TST suggests that the strategy of two-step TST specifically among contacts of active pulmonary TB cases for identify boosting subjects may not be worthwhile. This conclusion must be taken with caution because the small number of subjects followed.

## Conclusion

The low number of contacts with boosting and the difficulty in distinguishing boosting from TST conversion in the second TST suggests that the strategy of two-step TST testing among contacts of active TB cases may be not worthwhile. However, this conclusion must be taken with caution because the small number of subjects followed.

## Competing interests

The author(s) declare that they have no competing interests.

## Authors' contributions

CGS participated in the collection of data and in the analysis and interpretation of data. ARN performed the analysis and interpretation of data, as well as the revision of the manuscript. JRLS participated of the conception of the study and critically revised the manuscript. ALK participated of the conception of the study and critically revised the manuscript. MCC reviewed charts, collected data, organized the data base and participated of the analysis and interpretation of data. FCQM participated of the conception of the study and supervised the data collection and management. MBC participated of the conception and design of the study, analysis and interpretation of data and drafting the manuscript. All authors read and approved the final manuscript.

## Pre-publication history

The pre-publication history for this paper can be accessed here:


